# Female sexual medicine: an assessment of medical school curricula in a major United States city

**DOI:** 10.1093/sexmed/qfad051

**Published:** 2023-09-14

**Authors:** Nicolette Codispoti, Olivia Negris, Monica C Myers, Anna Petersen, Elsa Nico, Jennifer P Romanello, Rachel S Rubin

**Affiliations:** Stritch School of Medicine, Loyola University Chicago, Maywood, IL 60153, United States; Rush Medical College, Rush University, Chicago, IL 60612, United States; Chicago Medical School, Rosalind Franklin University of Medicine and Science, North Chicago, IL 60064, United States; Rush Medical College, Rush University, Chicago, IL 60612, United States; University of Illinois College of Medicine at Chicago, Chicago, IL 60612, United States; Rush Medical College, Rush University, Chicago, IL 60612, United States; Medstar Georgetown University Hospital, Department of Urology, Washington, DC 20007, United States

**Keywords:** female sexual dysfunction, medical education, curricular assessment

## Abstract

**Background:**

Although approximately 41% of women experience sexual dysfunction, limited education on female sexual medicine (FSM) in medical school results in underpreparedness among physicians when addressing these bothersome conditions.

**Aim:**

This study aims to evaluate the extent to which FSM is represented in medical education by examining current preclinical and clinical curricula.

**Methods:**

Preclinical curriculum materials on female sexual anatomy, physiology, and pathology, as well as obstetrics and gynecology clinical materials (syllabi, lecture materials, and supplemental resources), were collected from medical schools in the Chicago area. We utilized previous literature to identify specific components of medical school content to evaluate.

**Outcomes:**

Upon reviewing each institution’s curricula, we evaluated materials for topic saturation and assessed goals of each syllabus in terms of required content.

**Results:**

Curriculum materials were collected from 7 medical schools. In the preclinical assessment, 1 institution identified all anatomic components of the clitoris in our review, 4 discussed the physiology of the female orgasm, 3 highlighted the prevalence and epidemiology of female sexual dysfunction (FSD), 3 addressed treatments for FSD, and 1 instructed a genitourinary physical exam specific to assessing FSD. When assessing obstetrics and gynecology clinical materials, 5 institutions included topics related to FSM. Of these, only 1 institution had corresponding required synchronous clerkship time dedicated to these topics as a 1-hour lecture, in addition to an optional online training to third-year clinical students in comprehensive sexual history–taking practices, including screening for FSD. One other institution offered supplemental case-based gynecology modules including vulvovaginal diseases and chronic pelvic pain, though sexual pleasure, arousal, and libido were not included.

**Clinical Implications:**

The results of this study highlight the need for the inclusion of standardized curricula related to FSM in medical education to equip future physicians to treat patients with sexual dysfunction.

**Strengths and Limitations:**

The strengths of this study include that it is the first of its kind to complete a comprehensive review of FSM curricula at a cohort of undergraduate medical institutions. Its limitations include a small sample size of 7 medical schools limited to 1 geographical area.

**Conclusion:**

Our focused needs assessment of medical schools in the Chicago area reveals inconsistencies in outlined institution-specific course goals related to FSM and thus highlights the need for restructuring the curricula to prepare future physicians to recognize and treat patients with sexual dysfunction.

## Introduction

Sexual health is defined as a state of physical, mental, and emotional well-being related to sexuality.^1^ It has been well established in the literature that sexual health is impacted by many health conditions.^2-5^ However, physicians avoid discussing sexual health with their patients due to inadequate training that results in lack of comfort and clinical knowledge as well as limited time during office visits.^6^^,^^7^

Sexual health education in the United States peaked in 1997, when 95% of medical schools required some sexual health instruction.^8^ This number has since been decreasing, and only half of United States medical schools in 2018 required formal instruction in sexual health.^9^^,^^10^ The current curricula are not standardized and mostly consist of anatomy, physiology, and sexually transmitted infections (STIs), while excluding sexual dysfunction and sexual history taking.^6^^,^^9^^,^^11^ As a result, medical students have reported dissatisfaction with the quality and scope of their training and are not comfortable treating patients with sexual dysfunction.^6^^,^^8^^,^^9^^,^^11^ Research suggests that students believe that their role as physicians should include gathering a sexual history and treating sexual dysfunction, but they report not feeling equipped to do so.^5^ Several medical schools in the United States, Europe, and Brazil have recently created sexual medicine curricula that have increased students’ comfort taking sexual histories.^9^^,^^12^^,^^13^ However, these curricula have not been universally adopted and are not comprehensive.

One area of sexual health that is particularly lacking representation in medical education is female sexual dysfunction (FSD). FSD is a general term but is loosely defined as a problem during sexual response that reduces or inhibits satisfaction during sexual activity and causes distress.^14^ It can be subcategorized as disorders relating to libido, arousal, orgasm, or genitopelvic pain.^14^^,^^15^ Unfortunately, FSD is very common; one study found that 41% of women experience FSD. Another study found that 31% of young women experienced dysfunction that hindered their sexuality and 21% of young women experienced pain during intercourse “sometimes” or “often,” compared with only 4% of men.^16^^,^^17^ Studies also show that women who experience chronic vulvar pain are unlikely to seek medical care due to fear of stigma and distrust of physicians, and that those who do seek care are more likely to feel stigmatized by doctors.^17^^,^^18^

Despite FSD being both common and disruptive to overall health and wellbeing, female sexual medicine (FSM) has been extremely underresearched, underdiagnosed, undertreated, and undertaught. Because sexual medicine is not a unique specialty or subspecialty, or limited to one body system, all physicians should be educated and trained in sexual health and taking a sexual history. Studies have demonstrated that up to 62.3% of resident physicians in obstetrics and gynecology (OBGYN), urology, psychiatry, and endocrinology received no training in sexual medicine.^19^ All physicians ought to be trained in these topics during undergraduate medical education, as they are likely to encounter patients with sexual health concerns or dysfunction during their careers.

The aim of this needs assessment is to evaluate both the preclinical and clinical curricula of the 7 Chicago area medical schools and establish which aspects of female sexual anatomy, physiology, pathology, history taking, physical exam, and treatment options are included.

## Methods

This study was a review of both preclinical and clinical curricular materials from all 7 medical schools, including 6 allopathic institutions and 1 osteopathic institution, in a major United States city. Our institutions were selected based on author affiliation with a student-run organization based in Chicago, Illinois.

Materials were collected via students and faculty responsible for either the preclinical course on sex and reproduction or the third-year OBGYN clerkship at each academic institution. The OBGYN clerkship was selected as the focus for our study as a preliminary review of the other core clerkships (medicine, surgery, family medicine, pediatrics, psychiatry) at 3 institutions failed to include any sexual medicine education in curricular materials. As only institutional-level information was evaluated, with no collection of personal identifying information, this study was deemed Institutional Review Board exempt.

### Preclinical assessment

Preclinical content included syllabi, lecture materials, and corresponding supplemental resources related to anatomy, physiology, and pathology of the genitourinary tract. To standardize our needs assessment, we conducted an initial literature search to identify specific components of medical school course material to evaluate in our review. We categorized concepts based on anatomy, physiology, epidemiology, pathophysiology, physical exam, and treatment, a framework that aligns with how many topics are covered in medical school and recent sexual health competencies developed by Bayer et al.^20^ Upon reviewing each institution’s preclinical educational materials, we assessed content related to FSM and evaluated them based on topic saturation.

### Clinical assessment

Clinical content, including OBGYN clerkship syllabi, lecture materials, and supplemental resources, were also collected and assessed for inclusion of FSM. To standardize our clinical curriculum needs assessment, we limited the content reviewed to lectures and resources provided by the different academic institutions to students during their OBGYN rotation. Upon review of each institution’s clerkship materials, we assessed the learning objectives identified in each syllabus and evaluated the required learning content (including lecture schedules and online modules) for rotating students.

## Results

### Preclinical assessment

Didactic materials were collected from all 7 medical schools in the Chicago area. Due to the unique curricular design throughout undergraduate medical education, each institution incorporated components of interest differently. Institutions addressed these topics in courses such as anatomy, physiology, endocrinology, or others that included the study of the female reproductive tract. For this reason, a different number of educational materials were reviewed from each institution, although our saturation analysis strategy remained the same.

In the preclinical assessment, specific aspects of clitoral anatomy were reviewed. The number of institutions that identified each of the following areas of the clitoris were as follows: glans (6 of 7), corona (1 of 7), clitoral hood (2 of 7), corpus cavernosa (6 of 7), corpus spongiosa (4 of 7), crus (6 of 7), bulb (6 of 7), and clitoral neurovasculature (5 of 7). Only 1 institution discussed all the aforementioned anatomical parts of the clitoris.

In addition, 4 of 7 institutions discussed the physiology of the female orgasm, 3 of 7 institutions highlighted the prevalence and epidemiology of FSD, 3 of 7 institutions included information on treatment for FSD, and only 1 institution taught a genitourinary physical exam specific to assessing FSD, including external, internal, and pelvic floor assessment.

We also assessed whether students were taught how to take a thorough sexual history. Often, the training that medical students receive regarding a sexual history are limited to inquiring about partners, practices, protection from STIs, history of STIs, and pregnancy plans. When evaluating whether students were taught how to ask about more nuanced sexual health information, we found that 6 of 7 institutions reviewed sexual function and/or dysfunction, 2 of 7 institutions reviewed pleasure, and 2 of 7 institutions reviewed satisfaction.

### Clinical assessment

When assessing clinical materials, 5 of 7 institutions dedicate 6 weeks to the core OBGYN clerkship, while 2 others dedicate 5 weeks and 4 weeks, respectively. On assessment of the aims specified in the clerkship syllabi, 5 of 7 institutions included topics related to FSM. Of these, only 1 institution had corresponding required synchronous clerkship time dedicated to these topics. This was fulfilled as a 1-hour-long lecture. The same institution was the only one to offer training to third-year clinical students in comprehensive sexual history–taking practices, including screening for FSD. The format for this was an optional online module for students to complete independently outside of dedicated clerkship time.

Additionally, one other institution offered supplemental optional case-based gynecology modules, which included topics such as vulvovaginal diseases and chronic pelvic pain, though sexual pleasure, arousal, and libido were not discussed.

**Figure 1:**
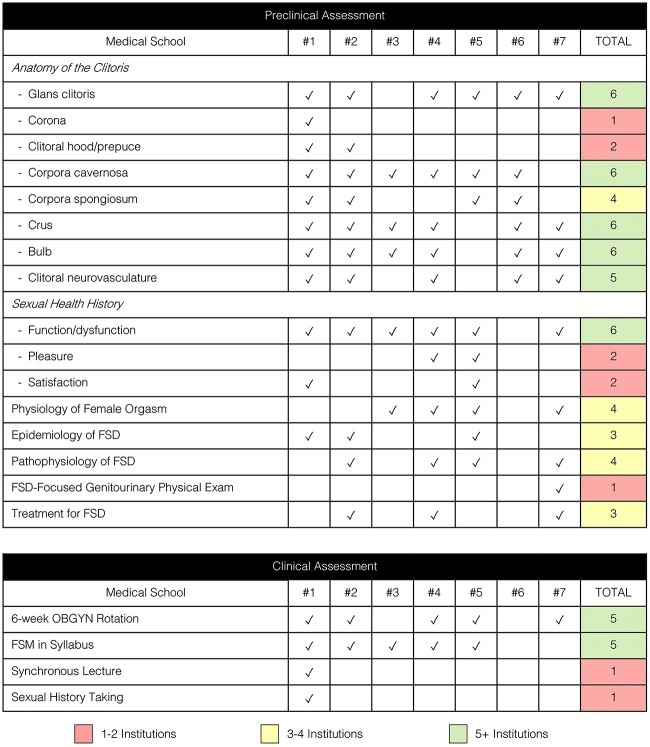
Preclinical and clinical assessment.

Our results from our preclinical and clinical assessment, stratified by institution and components of interest, can be found in Figure 1.

## Discussion

It has been well-documented that sexual dysfunction is pervasive and compounded by barriers to receiving appropriate care, such as stigma and lack of physician training in comprehensive sexual health^17^^,^^18^ As previous work acknowledges that sexual health training in medical school is lacking, particularly when it comes to FSM topics, this focused needs assessment of both allopathic and osteopathic medical schools in the Chicago area quantifies and explores the details of curricular shortcomings.^6^^,^^8^^,^^9^^,^^11^

### Preclinical assessment

The preclinical needs assessment specifically revealed incomplete instruction on clitoral anatomy, as only 1 institution identified all aspects of the clitoris delineated for this study within its didactic components. If physicians in training do not have the opportunity to become proficient in this crucial structure of female anatomy, physicians will continue to be unable to appropriately address the concerns of their patients. Surgeons that operate within the pelvis, including oncologic, gynecologic, urologic, colorectal, and plastic surgeons, may also leave patients vulnerable to suboptimal health outcomes or even injury if they receive inadequate education on clitoral anatomy in medical school.

An additional area of medical training that was identified as requiring standardization was sexual health history taking. While all institutions instructed students to inquire about partners, practices, protection from STIs, history of STIs, and pregnancy plans, only 2 institutions instructed students to inquire about pleasure and satisfaction. By neglecting to teach future physicians how to take a complete sexual history beyond basic reproductive health, patients may feel uncomfortable bringing concerns related to libido, arousal, orgasm, and pain to their providers due to fear that they are not interested or not trained to address these concerns. Medical students also play a critical role in gathering a history from patients during clinical rotations; therefore, it is imperative to teach trainees these skills on comprehensive sexual health history taking before entering the wards.

Our study found that only 1 institution taught a genitourinary physical exam specific to assessing FSD, including external, internal, and pelvic floor assessment. Evaluation of patients presenting with genitourinary symptoms is a core component of a comprehensive pelvic exam. Without knowledge of the skills required to assess the entire pelvis, accurate diagnosis and management for patients presenting with these issues may not be possible. In addition, as only 3 institutions included information on treatment for FSD, current aspects of preclinical curricula related to FSM are not adequate to prepare future physicians to provide treatment recommendations that are key to overall health and wellbeing.

### Clinical assessment

The clinical needs assessment reviewed institution-specific clerkship goals set forth by syllabi and the required content made available to students. Our assessment identified inconsistencies within these materials, highlighting the need to restructure clinical curricula to include topics related to FSM.

Clinical clerkships in OBGYN typically range from 4 to 6 weeks in duration. Of the 7 institutions, only 1 had a required synchronous lecture dedicated to FSM. From our review, it is not clear why only 1 institution set aside dedicated time to review sexual health. We hypothesize that individual faculty members and/or departmental interest in this important area may be a driving factor for its inclusion. As discussed previously in the preclinical assessment, medical students must be exposed to topics in FSM to work toward proficiency and confidence in these areas, apply this knowledge in real-life clinical encounters, and support patient concerns.

All medical schools should include early, standardized education related to sexual medicine and dysfunction to properly train future physicians.^8^^,^^12^^,^^21^ Without this education, medical trainees will continue to lack the foundational knowledge of these concepts unless this content is recognized as a necessary part of both the preclinical and clinical years in undergraduate medical education. As students also typically rely on third-party resources to acquire knowledge in preparation for both clinical and board exams, FSM content must also be considered testable material by medical institutions including the National Board of Medical Examiners and United States Medical Licensing Examination. This would lead to adoption of content areas in sexual health not only by medical education faculty and third-party study resources, but also by students who are actively preparing for these pivotal examinations that take place during medical school.

There are a few limitations of our study. We utilized students and designated faculty to gather the curricular materials, and it is possible that not all aspects of the sexual health curriculum were included in our evaluation. Clinical clerkship content can be highly variable and susceptible to changes throughout the academic year, whereas preclinical content usually does not change as quickly. Therefore, it is possible that content changes or additions, particularly to the clinical curricula, were made without the knowledge of reviewers. We recognize that informal teaching may also occur during student rotations and would not be captured in the formal curricular materials. However, our study aims to highlight that formal teaching of these topics must be standardized to adequately prepare all trainees to manage sexual health concerns. As we only completed a preliminary review of core clerkship materials at 3 institutions, FSM content may have been presented in other required clinical rotations at the remaining institutions, although the likelihood of this is low. Other clinical rotations, including urology, are not required, and thus cannot be depended on to universally train future physicians regarding FSM. This would have also resulted in an omission of FSM materials for inclusion in this review.

An additional limitation of this study is that the curricular review focused on one geographic area. Data from our selected medical schools can be utilized to provide insight on how sexual health curriculum may be structured at other institutions throughout the country. The Chicago area has the second highest number of medical schools compared with any other city in the United States. The institutions located here represent a diverse range of academic rigor, admissions standards, and philosophy. Specifically, our cohort includes 5 institutions with academic hospitals, 1 state institution, 1 religiously affiliated institution, and 1 institution with osteopathic designation. Four of the medical schools are in urban settings and 3 are in suburban settings. In addition, per the publicly available match data, these Chicago area medical schools collectively graduate approximately 1,200 new physicians annually who subsequently travel across the United States to pursue residency training and careers. Our results suggest similar trends may exist at medical schools throughout the country; thus, the existing educational content in our cohort of medical schools is relevant to other institutions and programs across the country.

As this study is the first to intimately assess the current curricular content that surrounds both FSM and FSD, our findings are a starting point from which we can begin to understand what structured content is being disseminated to medical students. Additional research is needed to fully evaluate the inclusion of FSM in medical education across the United States and to explore regional and institution-based differences. We also feel that it is important to review topics in FSM during undergraduate medical education, as only programs with relevance to sexual health, either medically or surgically, will likely explore these topics during graduate medical education, or during residency.

Future directions include proposing curricular recommendations to these institutions with the goal of standardizing and enhancing medical student exposure to FSM topics. Although recommendations may not entirely lead to adoption of this material within the medical school curriculum, it can help provide the framework in doing so. Additional aims include evaluating institutional and national-level examination questions to see how FSM topics are, or are not, being tested.

## Conclusion

Our needs assessment of medical schools within the Chicago area highlights the need to standardize both preclinical and clinical materials to enhance student education, exposure, and preparation as it relates to FSM. Our goal is to prepare all future physicians to approach patients with sexual health concerns during and after medical school training. If we continue the status quo of FSM education as it stands today, we are doing an injustice to our patients who deserve clinicians that can have a baseline discussion of sexual health, with the gold standard of providing a comprehensive sexual health evaluation based on specialty or referring to a qualified expert in the field.

## Funding

This research did not receive any specific grant from funding agencies in the public, commercial, or not-for-profit sectors.

## Conflict of interest

None declared.
